# Matriptase shedding is closely coupled with matriptase zymogen activation and requires de novo proteolytic cleavage likely involving its own activity

**DOI:** 10.1371/journal.pone.0183507

**Published:** 2017-08-22

**Authors:** Chun-Che Tseng, Bailing Jia, Robert Barndt, Yayun Gu, Chien-Yu Chen, I-Chu Tseng, Sheng-Fang Su, Jehng-Kang Wang, Michael D. Johnson, Chen-Yong Lin

**Affiliations:** 1 Lombardi Comprehensive Cancer Center, Department of Oncology Georgetown University, Washington DC, United States of America; 2 Department of Gastroenterology, Henan Provincial People’s Hospital, Zhengzhou, China; 3 Department of Biochemistry National Defense Medical Center, Taipei, Taiwan; 4 School of Medicine National Defense Medical Center, Taipei, Taiwan; Russian Academy of Medical Sciences, RUSSIAN FEDERATION

## Abstract

The type 2 transmembrane serine protease matriptase is involved in many pathophysiological processes probably via its enzymatic activity, which depends on the dynamic relationship between zymogen activation and protease inhibition. Matriptase shedding can prolong the life of enzymatically active matriptase and increase accessibility to substrates. We show here that matriptase shedding occurs via a de novo proteolytic cleavage at sites located between the SEA domain and the CUB domain. Point or combined mutations at the four positively charged amino acid residues in the region following the SEA domain allowed Arg-186 to be identified as the primary cleavage site responsible for matriptase shedding. Kinetic studies further demonstrate that matriptase shedding is temporally coupled with matriptase zymogen activation. The onset of matriptase shedding lags one minute behind matriptase zymogen activation. Studies with active site triad Ser-805 point mutated matriptase, which no longer undergoes zymogen activation or shedding, further suggests that matriptase shedding depends on matriptase zymogen activation, and that matriptase proteolytic activity may be involved in its own shedding. Our studies uncover an autonomous mechanism coupling matriptase zymogen activation, proteolytic activity, and shedding such that a proportion of newly generated active matriptase escapes HAI-1-mediated rapid inhibition by shedding into the extracellular milieu.

## Introduction

The type 2 transmembrane serine protease matriptase is expressed primarily in epithelial cells [1,2] and required for the maintenance of epithelial integrity and function [3]. Dysregulation of matriptase may contribute to carcinogenesis and cancer progression and other diseases [4–8]. Matriptase is synthesized as a zymogen, which can be converted, via an autoactivation mechanism, into an active enzyme with potent trypsin-like activity [9–12]. The pathophysiological roles of matriptase have been attributed to its proteolytic activity by activating and/or processing its downstream substrates [6,13–15]. In addition to the autoactivation mechanism, matriptase proteolytic activity is tightly regulated by endogenous protein protease inhibitors, including the two integral membrane Kunitz-type serine protease inhibitors, hepatocyte growth factor activator inhibitor (HAI)-1 and HAI-2 [16–18]. Although less obvious and not as well characterized, the shedding from cells of the matriptase extracellular domains, which includes the catalytic domain, represents an important mechanism governing the temporal and spatial aspects of matriptase proteolytic activity. The rapid inhibition of nascent active matriptase by HAI-1 following matriptase zymogen activation renders cell-associated active matriptase a short-lived species [19,20]. The shedding into the extracellular milieu not only prolongs the half-life of active matriptase but also provides expanded access for matriptase to its potential substrates [21]. The shedding into the extracellular milieu may be particularly important for the role of matriptase in cancer. Within the tumor microenvironment, the shed active matriptase can activate its cancer-related substrates, including urokinase, HGF, and PDGF-D in the pericellular space, providing an important mechanism for cancer cells to interact with stromal cells [22–24].

Matriptase shedding involves the release of the enzyme from the cell surface in soluble form and is thought to involve proteolytic cleavage. This is analogous to the process of ectodomain shedding which participates in the regulation of a diverse set of proteins, including extracellular matrix components, cell adhesion molecules, growth factors, cytokines, and membrane receptors [25,26]. Protein ectodomain shedding can be induced by various stimuli including phorbol esters, cytokines, and growth factors. Members of metalloprotease family, including the disintegrin and metalloproteases (ADAMs) and the matrix metalloproteases frequently participate in ectodomain shedding [27]. Characterization of the shedding of epithin, the murine matriptase ortholog, suggests that tumor necrosis factor-α converting enzyme (TACE/ADAM17) mediates the cleavage of shedding when thymic epithelial cells are treated with TGF-β [28]. Epithin shedding can be also induced by phorbol myristate acetate and inhibited by a pan metalloprotease inhibitor (Kim et al., 2005), further supporting the role of the canonical ectodomain shedding in the regulation of matriptase function.

The mature matriptase zymogen is composed of two fragments resulted from an autolytic cleavage at Gly-149 within the SEA domain, a process which likely occurs during protein synthesis and maturation in the endoplasmic reticulum (ER) and Golgi apparatus [10,29]; such autolytic cleavage is common in SEA domain-containing proteins, including the mucins [30]. Noncovalent interaction between the N-terminal fragment (NTF) and the C-terminal fragment (CTF) of the SEA domain are, however, of sufficient affinity to hold the two matriptase chains together (matriptase NTF; Met1-Gly149, and matriptase CTF; Ser150-Val855). Thus, the bulk of the matriptase extracellular domain is tethered to the plasma membrane by the transmembrane domain through the non-covalent interactions within the cleaved SEA domain. Sequencing of matriptase CTF purified from human milk, identified Ser-190 or Thr-205 as the N-terminal amino acids [31], suggesting that proteolytic cleavage between Lys-189 and 190-Ser and/or at Lys-204 and Thr-205 could either be directly involved in, or occur subsequent to matriptase shedding. Proteases involved in this cleavage, therefore, must prefer a Lys residue at the P1 site and so are most likely serine proteases with trypsin-like activity. Thus if these N-termini are directly generated during matriptase shedding, the observed cleavage sites are not consistent with shedding being mediated by the proteolytic activity of TACE/ADAM17, the cleavage preference of which is an alanine residue at the P1 site and valine residue at the P1’ site [32]. This paradox led us in the current study, to revisit this cardinal mechanism governing matriptase shedding. Our study indicates that matriptase shedding involves cleavage at Arg-186, which is temporally coupled with, and depends on, matriptase zymogen activation. The rapid kinetics of shedding and its coupling with matriptase zymogen activation allows a proportion of the nascent active matriptase to escape from rapid inhibition by HAI-1 and to be shed into the extracellular milieu where it has access to substrates in the pericellular environment.

## Materials and methods

### Cell cultures

Human breast cancer cell lines SK-BR-3, MCF7, and MDA-MB-468 (ATCC, Manassas VA) were cultured in a modified Improved Minimum Essential Medium (IMEM) supplemented with 10% fetal bovine serum (FBS). 184 A1N4 human mammary epithelial cells (a gift from M. R. Stampfer, UC Berkeley) [33] were cultured in IMEM supplemented with 0.5% FBS, 5 μg/ml recombinant human insulin (rh-insulin—Invitrogen, Carlsbad, CA), 5 μg/ml hydrocortisone (Sigma-Aldrich, St. Louis, MO), and 10 ng/ml recombinant human epidermal growth factor (rhEGF) (Promega, Madison, WI). Caco-2 (ATCC, Manassas VA) human colorectal adenocarcinoma cells, HaCaT human keratinocytes (CLS Cell Lines Service GmbH, Eppelheim Germany), and 293T human embryonic kidney (HEK) cells (ATCC, Manassas VA) were cultured in Dulbecco's Modified Eagle's Medium (DMEM) supplemented with 10% FBS. LNCaP (ATCC, Manassas VA) prostate cancer cells were cultured in RPMI-1640 medium supplemented with 10% FBS. All cells were incubated at 37°C in a humidified atmosphere with 5% CO2. To collect the secreted proteins, the cells were cultured in their respective medium, containing no FBS for 24 hrs. The conditioned medium was collected and concentrated using centrifugal filter units (Millipore, Billerica, MA).

### Expression and purification of matriptase N-terminal fragment for monoclonal antibody generation

Recombinant protein antigen consisting of the matriptase N-terminal fragment (AA 1–452), with a FLAG tag at the amino (N) terminus and a His tag at the carboxyl (C) terminus, was prepared for use as an immunogen for the generation of monoclonal antibodies by hybridoma fusion. DNA encoding the first 452 amino acids of matriptase was amplified with the following PCR primers: forward primer: 5’- ACCGGATCCATGGACTACAGGGACGACGATGACAAAATG; reverse primer: 5’- TGGCTCGAGTTAGTGATGATGGTGATGATGGTGATGTGG-3’. An adenoviral expression construct containing the PCR product, was prepared using the pAd/CMV/V5-DEST™ Gateway® Vector Kit (Life Technologies, Carlsbad, CA). The construct was packaged following the manufacturer’s instructions and 184 A1N4 human mammary epithelial cells were infected and used to express the matriptase fragment. Cell pellets were collected and lysed in 20 mM Tris buffer containing 1% Triton X-100. The recombinant matriptase fragment was purified from the cell lysate by ionic exchange chromatography using a DEAE-Sepharose FF column followed by immunoaffinity chromatography using anti-FLAG mAb-Sepharose beads. The proteins eluted from the immunoaffinity column were resolved by SDS-PAGE, and the gel stained with Coomassie Brilliant Blue. Bands of approximately 50- and 35-kDa were sliced from the gel and the proteins therein subjected to in-gel trypsinization followed by mass spectrometry-based proteomic protein identification using the services of the Proteomics & Metabolomics Shared Resource, Lombardi Comprehensive Cancer Center, Georgetown University. The purified protein was used as an immunogen to generate matriptase N-terminal fragment mouse mAbs, including PS6 and PS7, by conventional immunization and hybridoma fusion methods, under a protocol approved by the Georgetown University Institutional Animal Care and Use Committee. Through a combination of targeted mutation and deletion studies (not shown) the location of the epitopes recognized by mAbs PS6 and PS7 have been mapped to somewhere in the matriptase intracellular domain between M1 and R54.

### Monoclonal antibodies

The mAb M24, the generation and characterization of which has been described previously, was used to detect human matriptase C-terminal fragment, which contains the serine protease domain [16]. The mAbs PS6 and PS7 were used to detect the matriptase N-terminal fragment. The M19 mAb was used to detect human HAI-1-containing species [16]. Matriptase mAbs PS6 and M24 and HAI-1 mAb M19 were also immobilized to Sepharose beads at 5 mg/ml gel, according the manufacturer’s instruction (GE Healthcare Life Science, Marlborough, MA).

### Western blotting

Cells were lysed in phosphate buffered saline (PBS) containing 1% Triton X-100 and 1 mM 5,5’-Dithio-bis-(2-Nitrobenzoic Acid) (DTNB), which was used to prevent cleavage of disulfide linkages [34]. Protein samples were diluted with 5x SDS sample buffer containing no reducing agent and incubated at room temperature for 5 min. Proteins were resolved by either 7.5% SDS-PAGE or Tricine-SDS-PAGE [35] for analysis lower molecular weight proteins, such as the matriptase N-terminal fragment, and then transferred to nitrocellulose membranes. Immunoblot analysis of the membranes was conducted with the indicated mAbs followed by HRP-conjugated secondary antibodies and visualized using the Western Lightening Chemiluminescence Reagent Plus (Perkin-Elmer, Boston, MA) and x-ray film or Amersham Imager 600 (GE Healthcare Life Sciences, Marlborough, MA).

### Immunodepletion

Immunodepletion was carried out by mixing cell lysates (200 μl) with 15 μl of the indicated mAb-conjugated Sepharose beads in 600 μl micro centrifuge tubes that were rotated end over end in a cold room for 2 hours. The supernatant was collected as the immunodepletion fraction after the beads were pelleted by centrifugation.

### Induction of matriptase zymogen activation

In order to induce matriptase zymogen activation in living cells, the cells were washed with PBS three times and incubated with 150 mM phosphate buffer pH 6.0 at room temperature for 20 min, as previously described [19]. In order to induce matriptase zymogen activation in the cell-free setting, the cells were homogenized in PBS using a Dounce homogenizer. The insoluble fractions of the cell homogenates were then pelleted by centrifugation at 10,000 rpm for 2 min using an Eppendorf table-top centrifuge. The resultant pellets were re-suspended in 150 mM phosphate buffer pH 6.0 and incubated at room temperature for 20 min, as previously described [36]. The phosphate buffer conditioned by exposure to the cells in culture, or the insoluble homogenized cell pellets, were collected and retained as the shed fraction.

### Site-directed mutagenesis

A full length cDNA clone of human matriptase in pcDNA3.1 (Invitrogen Corporation, Carlsbad, CA) was used to prepare site-directed or deletion mutants. Sequencing of this cDNA revealed a silent mutation at nucleotide 597 from a C to T, as compared to the published sequence (GenBank AF118224). Site-directed mutagenesis was conducted with the QuikChange Site Directed Mutagenesis Kit (Stratagene, La Jolla, CA) using primers containing the desired nucleotide changes according to the manufacturer’s protocol. Preparation of the S805A matriptase mutant has been previously described [10]. For double mutations R186A/K189A, the primers 5’-CCCCCGCGGGCGGCCTCCTTGGCGTCCTTTGTGGTC-3' and 5'-GACCACAAAGGACGCCAAGGAGGCCGCCCGCGGGGG-3' were used. For K204A/R208A, the primers 5'-TTCCCCACGGACTCCGCAACAGTACAGGCGACCCAGGACAACAG-3' and 5'-CTGTTGTCCTGGGTCGCCTGTACTGTTGCGGAGTCCGTGGGGAA-3' were used. For R186A, the primers 5’-CCCCCGCGGGCGGCCTCCTTGAAGTCC-3’ and 5’-GGACTTCAAGGAGGCCGCCCGCGGGGG-3’ were used. For K189A, the primers 5’-CGGGCGCGCTCCTTGGCGTCCTTTGTGGTCAC-3’ and 5’-GTGACCACAAAGGACGCCAAGGAGCGCGCCCG-3’ were used. For K204A, the primers 5’-CTTTCCCCACGGACTCCGCAACAGTACAGAGGACCC-3’ and 5’-GGGTCCTCTGTACTGTTGCGGAGTCCGTGGGGAAAG-3’ were used (Integrated DNA Technologies, Coralville, IA). All site-directed mutations and deletion mutants were confirmed by DNA sequencing (Genewiz, Frederick MD).

### Transfection

Transient co-transfection of human matriptase (wild-type or mutant) and HAI-1 constructs were accomplished using Lipofectamine 2000 (Thermo Fisher Scientific, Waltham, MA) according to the manufacturer’s protocol. When doing single vs co-transfections, the total amount of DNA used with the transfection reagent was kept constant for each individual transfection by including empty vector pcDNA3.1 DNA, where appropriate.

## Results

### Generation of monoclonal antibodies targeting matriptase NTF

**Fig 1:**
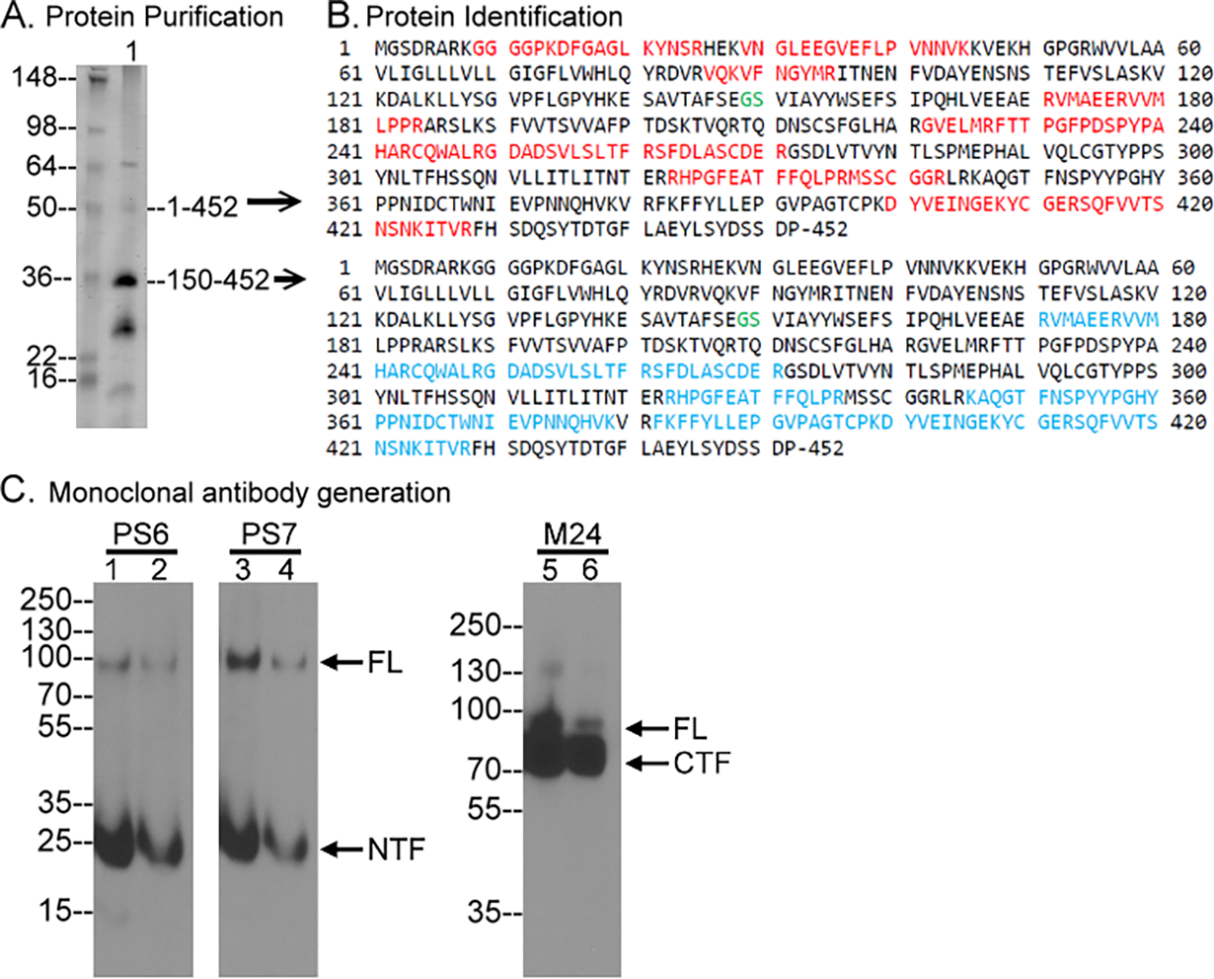
Generation of matriptase NTF monoclonal antibodies. *A*. Matriptase NTF was expressed and purified. The purified proteins were analyzed by SDS-PAGE and visualized by Coomassie Blue (lane 1). *B*. The 50- and 35-kDa protein bands were identified by in-gel trypsinization and tandem mass spectrometry (MS/MS). The identified tryptic peptides from the 50-kDa protein matching matriptase are indicated in red and from the 35-kDa protein in blue. The SEA domain cleavage site Gly149-Ser-150 (GS) in green is also indicated. *C*. Two different amounts of 184A1N4 human mammary epithelial cell lysate were analyzed by western blot under non-reducing and non-boiled condtions for matriptase species using the mAbs PS6 (lanes 1 and 2), PS7 (lanes 3 and 4) and M24 (lanes 5 and 6). FL stands for full-length, NTF for N-terminal fragment, and CTF for C-terminal fragment. The immunoblot data presented are representative examples of at least two independent experiments.

In order to study matriptase shedding, a new panel of matriptase mAbs were generated using recombinant matriptase N-terminal fragment as the immunogen (Fig 1A). The recombinant matriptase fragment contains amino acid residues 1 to 452, which includes the intracellular domain, the transmembrane domain, the SEA domain and the two CUB domains. Analysis of recombinant protein purified from 184A1N4 cells infected with the matriptase-NTF-Flag construct yielded a minor protein band of 50-kDa, two major bands of 36- and 25-kDa, and a minor band of 15-kDa. The size of the 50-kDa protein band was close to the calculated size of the recombinant NTF. The 18 tryptic peptides identified from analysis of the 50-kDa band matched to matriptase and were located on both sides of Gly149-Ser150: the SEA domain autolytic site (Fig 1B, upper panel, in red). The apparent size and the distribution of the tryptic peptides suggest that the 50-kDa band is the entire expressed recombinant matriptase species containing an intact SEA domain. The 36-kDa protein band is the C-terminal fragment of the recombinant matriptase, resulting from the autolytic cleavage of the SEA domain at Gly149-Ser150, based on the 17 tryptic peptides identified on analysis of that band matching to matriptase sequences between Gly-150 and Pro-452 (Fig 1B, lower panel, in blue). The ratio of the processed matriptase fragment (36-kDa) relative to the unprocessed form (the 50-kDa) indicates that the majority of the recombinant matriptase, as with the natural full-length matriptase, has undergone N-terminal processing by cleavage of the SEA domain.

The purified recombinant matriptase was used as an immunogen to generate monoclonal antibodies (mAbs) using hybridoma fusion methods. Screening of the hybridoma clones led to the identification of a panel of mAbs of which two mAbs (PS6 and PS7) were shown to recognize the matriptase N-terminal fragment (NTF) between aa 1–149 and were further characterized and used in the current study. Both of these matriptase N-terminal mAbs can detect 94-kDa full-length (FL) matriptase and the 25-kDa matriptase NTF (Fig 1C, lanes 1–4). In contrast, the matriptase mAb M24 detects both the 70-kDa C-terminal fragment (CTF) and full-length matriptase (Fig 1C, lanes 5 and 6).

### Tight association between the cleaved SEA domain fragments holds the matriptase NTF and CTF together on the cell membrane

**Fig 2:**
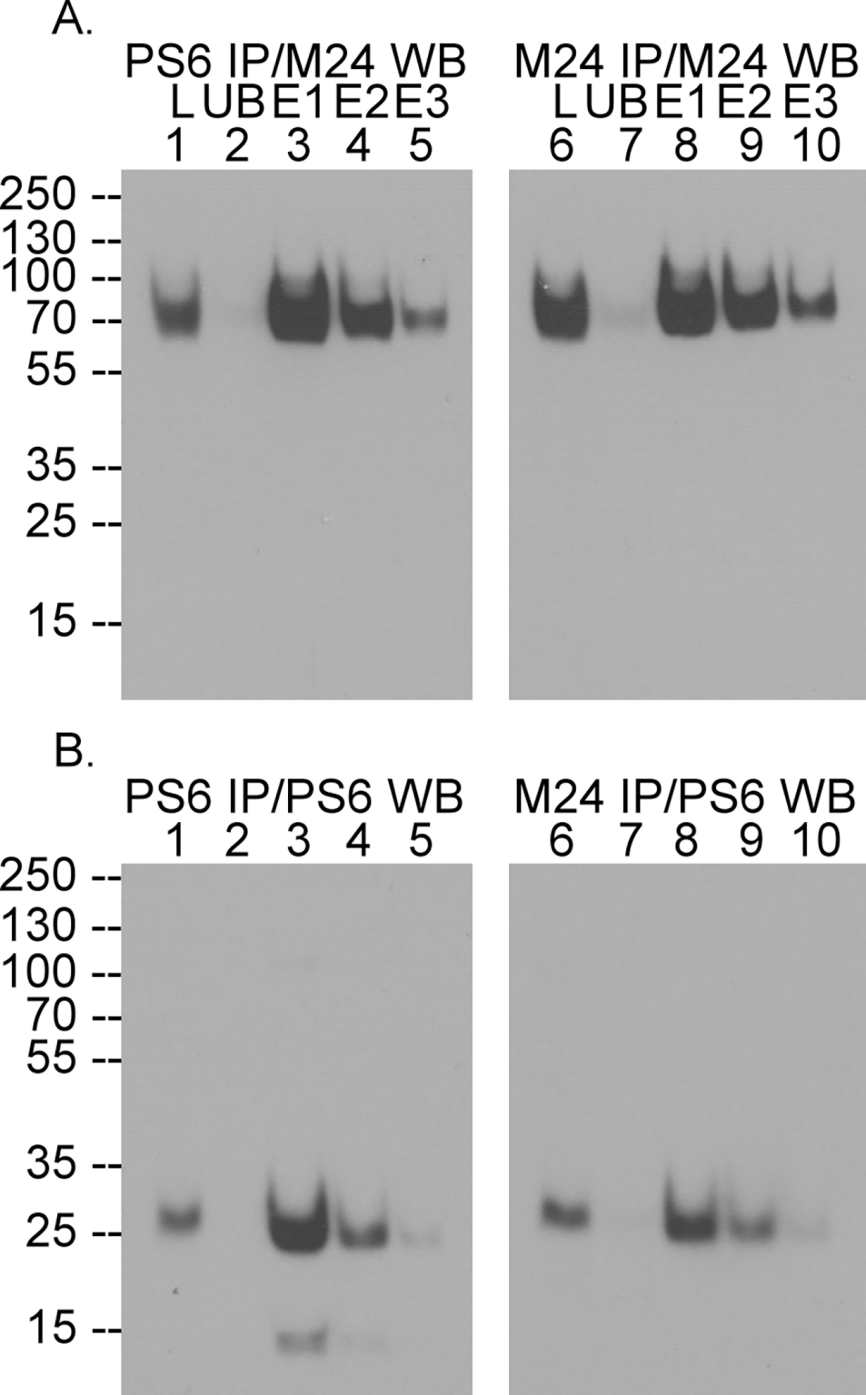
Matriptase is tethered on the plasma membrane in spite of the cleavage of the SEA domain. HaCaT human keratinocyte cells lysates (lanes 1, L) were incubated with the matriptase NTF mAb PS6-Sepharose (PS6 IP, lanes 1–5) or CTF mAb M24-Sepharose (M24 IP, lanes 6–10). The unbound fraction (lanes 2 and 7, UB) was collected as the immunodepletion fraction. The mAb-captured proteins were sequentially eluted into three fractions (lanes 3–5 and 8–10, E1-E3). These samples were analyzed by western blot under non-reducing and non-boiled condtions for matriptase CTF by the mAb M24 (*A*, M24 WB) and matriptase NTF by the mAb PS6 (*B*, PS6 WB). The immunodepletion-immunoblot data presented are representative examples of at least two independent experiments.

The association of the NTF and CTF of SEA domain cleaved matriptase on the cell membrane can be demonstrated by a combination of immunoprecipitation of matriptase NTF or CTF followed by immuoblot detection of matriptase CTF or NTF, respectively (Fig 2). When cell lysates were incubated with the NTF mAb PS6 immobilized on Sepharose, both the NTF (Fig 2B, comparing lane 2 with lane 1) and the CTF of matriptase (Fig 2A, comparing lane 2 with lane 1) were immunodepleted from the lysates. Analysis of the proteins released from the PS6 mAb-Sepharose beads by a pH 2.4 buffer demonstrates the presence of both the NTF and CTF of matriptase (Fig 2, lanes 3–5). Similarly, when the cell lysates were incubated with the matriptase CTF mAb M24-Sepharose, both matriptase CTF (Fig 2A, comparing lane 7 with lane 6) and matriptase NTF (Fig 2B, comparing lane 7 with lane 6) were immunodepleted. Again, both matriptase CTF and matriptase NTF are released from the M24 mAb-beads by a pH 2.4 buffer (Fig 2, lanes 8–10). These data suggest that while the SEA domain is cleaved at Gly149-Ser150, the bulk of the matriptase extracellular domains remains tethered on the lipid bilayer biomembrane via the strong non-covalent interaction within the cleaved fragments of the SEA domain.

### Matriptase shedding involves de novo proteolytic cleavage

**Fig 3:**
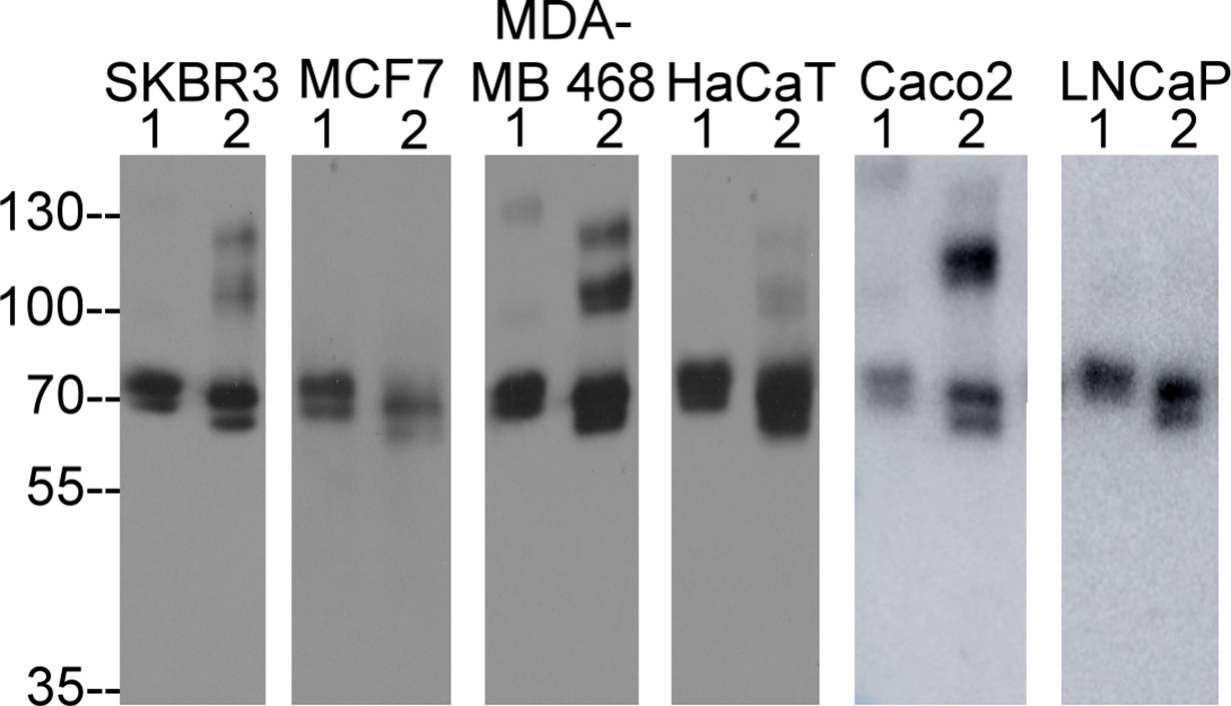
Matriptase shedding requires de novo proteolytic cleavages. Six different human cell lines, as indicated, were grown in the medium containing no serum for 24 hrs. The conditioned media were collected and concentrated. The cell lysates (lanes 1) and conditioned medium (lanes 2) were analyzed by western blot under non-reducing and non-boiled conditions for matriptase species using the matriptase mAb M24. The samples were analyzed by loading various sample volumes in order to clearly demonstrate the sizes of cell-associated and shed matriptase. The analyses of matriptase species in the cell lysates and conditioned media from cells was conducted at least two times, and representative data are presented.

Given the above, for matriptase to be shed from the cell surface, there would appear to be two possible mechanisms: 1) dissociation of the SEA domain fragments that result from the preexisting cleavage between Gly-149 and Ser-150, resulting in the shedding of a matriptase species with the same N-terminus (Ser-150) and the same size as the cell-associated counterpart or 2) dissociation via de novo proteolytic cleavage, resulting in a shed matriptase species with new N-terminus and different (smaller) size from the cell-associated counterpart. We therefore set out to compare the size of cell-associated matriptase to that of the shed form in order to determine which mechanism might be involved in matriptase shedding (Fig 3). Cell-associated matriptase zymogen was detected as a doublet of around 70-kDa with the upper band of the doublet being more intense in all of the six cell lines examined (Fig 3 lanes 1). The shed matriptase present in the conditioned medium collected from the cells was also detected as a doublet with the upper band more intense than the lower one (Fig 3, lane 2), but the size of both bands was noticeably smaller than seen for the cell-associated matriptase. The smaller size of the shed matriptase species supports the hypothesis that de novo proteolytic cleavage is involved in matriptase shedding.

### Matriptase shedding is temporally coupled with and dependent on matriptase zymogen activation

**Fig 4:**
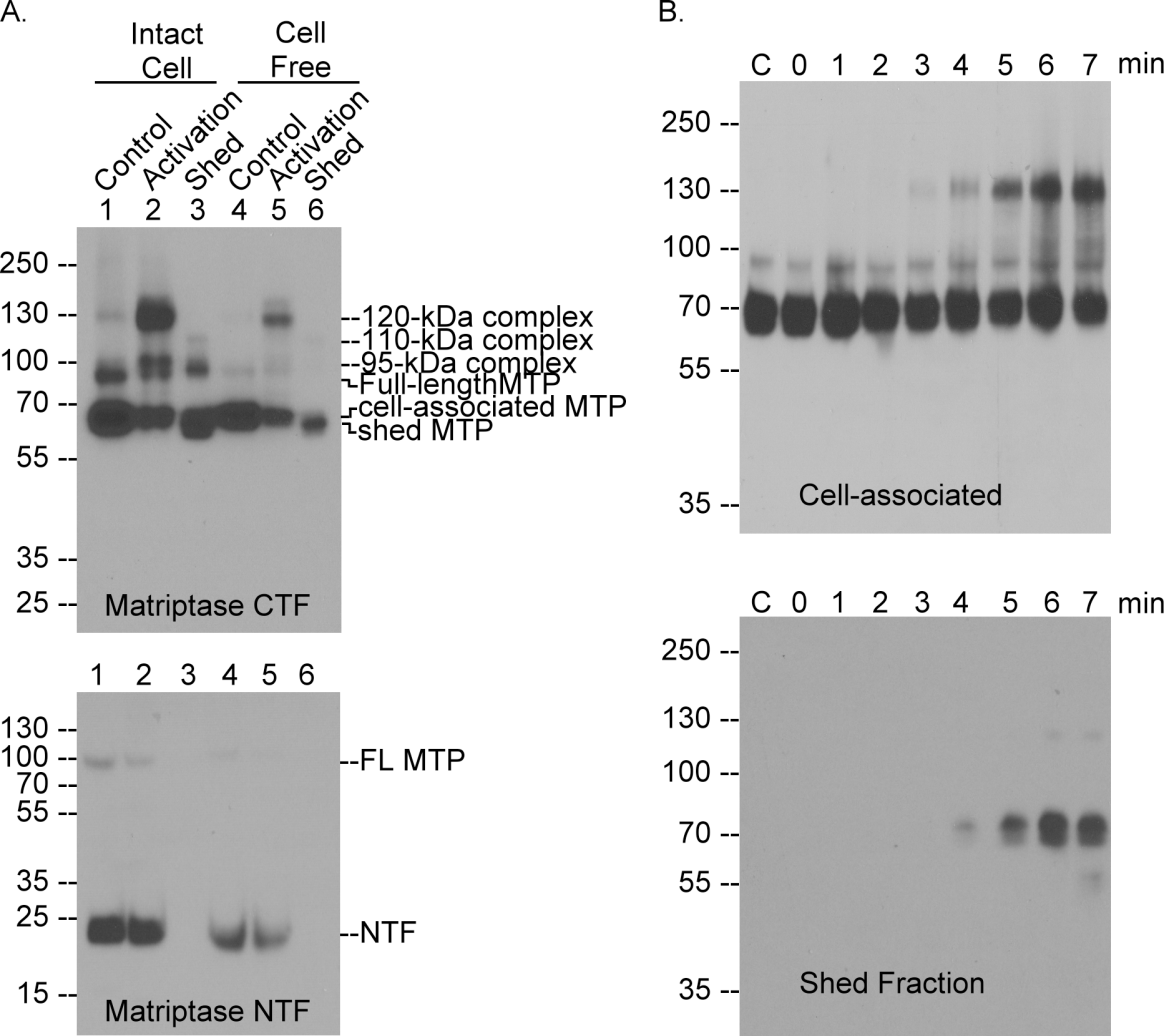
Matriptase shedding is temporally coupled with matriptase zymogen activation. *A*. LNCaP human prostate adenocarcinoma cells (lanes 1–3) or the insoluble fractions of the cells (lanes 4–6) were exposed to PBS as the non-activation control (lanes 1 and 4, Control) or phosphate buffer pH 6.0 for 20 min to induce matriptase zymogen activation (lanes 2 and 5, Activation). The conditioned buffer was collected as the shed fraction (lanes 3 and 6, Shed). The lysates and the conditioned buffer were analyzed by western blot for matriptase CTF using the mAb M24 (upper panel) and for matriptase NTF using the mAb PS6 (lower panel). Various matriptase (MTP) species were indicated, including the three matriptase-HAI-1 complexes with apparent masses of 120-, 110-, and 95-kDa, and full-length, cell-associated, and shed matriptase (MTP). *B*. HaCaT human keratinocytes were exposed to a phosphate buffer pH 6.0 for indicated time from 0 to 7 min or PBS as control (lanes C). The shed fractions were concentrated to the same volume as that of the cell lysate. Equal volume of the cell lysate (Cell-associated, upper panel) and the concentrated conditioned buffer (Shed fraction, lower panel) were analyzed by immunoblot under non-reducing and non-boiled conditions for matriptase species. The data presented are representative of that obtained in more than three independent experiments.

Previous studies indicate that matriptase shedding may be temporally coupled with matriptase zymogen activation. For example, the onset of matriptase zymogen activation and matriptase shedding can be observed within the same time frame: around 6 hours after the exposure of LNCaP prostate cancer cells to androgens [37]. Matriptase zymogen activation is also closely followed by matriptase shedding in human mammary epithelial cells when the cells are exposed to the lysophospholipid, sphingosine 1-phosphate [31,38]. The temporal relationship between matriptase zymogen activation and shedding was further investigated by comparing the minute-scale kinetics of matriptase zymogen activation and matriptase shedding using a model system, in which robust, and rapid matriptase zymogen activation is induced by exposure to a mildly acidic buffer (pH 6.0) (Fig 4). Acid-induced matriptase zymogen activation takes advantage of an unusual feature of matriptase regulation. Matriptase is synthesized as a zymogen and only acquires its full enzymatic activity upon cleavage at Arg-614 in the activation motif. While some other serine proteases with trypsin-like activity can activate matriptase, autoactivation via its intrinsic zymogen activity is thought to be the primary mechanism for matriptase zymogen activation [10]. Matriptase zymogen can undergo autoactivation at pH 7.4, though at a relatively low rate. Exposure of cells to a mildly acidic buffer, however, greatly accelerates the process of autoactivation [39]. Anchorage of matriptase on a lipid bilayer is required and appears to be sufficient for matriptase to undergo acid-accelerated zymogen activation [36] and indeed acid-induced matriptase zymogen activation can be observed using preparations of the insoluble fractions of cell homogenates [36] as well as in intact cells [19].

When LNCaP cells (Fig 4A, lanes 1–3) and the insoluble fraction of cell homogenates (Fig 4A, lanes 4–6) were incubated with phosphate buffer pH 6.0 for 20 min, matriptase zymogen activation was induced, as determined by the appearance of the 120-kDa activated matriptase-HAI-1 complex at the cost of the 70-kDa matriptase zymogen (Fig 4A, upper panel, comparing lane 2 with lane 1 or lane 4 with lane 5). When the conditioned buffer collected from the cells was analyzed for matriptase species, the “shed fraction” was found to be comprised of a 70-kDa species with a size slightly smaller than its cell-associated counterpart (Fig 4A, upper panel, comparing lane 3 with lane 2 or lane 6 with lane 5). Two bands comprised of matriptase-HAI-1 complexes of 110- and 95-kDa were also clearly detected in the shed fraction (Fig 4A, upper panel, lane 3). In addition, the matriptase NTF which contains the transmembrane domain, remains cell-associated before and after induction of robust matriptase zymogen activation (Fig 4A, lower panel), as expected. Collectively, the data generated with the acid-induced matriptase activation model system not only recapitulates the rapid inhibition of active matriptase by HAI-1 and the involvement of de novo proteolytic cleavage in matriptase shedding, but also suggests that matriptase shedding rapidly follows matriptase activation.

In order to establish the temporal relationship between matriptase shedding and matriptase zymogen activation, the kinetics of both events were compared using the pH 6.0-induced matriptase zymogen activation model (Fig 4B). The onset of matriptase zymogen activation, as determined by the appearance of cell-associated activated matriptase complex with HAI-1, began as few as 3 minutes post acid exposure and the process proceeded rapidly (Fig 4B, upper panel). The onset of matriptase shedding, as determined by the appearance of matriptase in the conditioned buffer, began as few as 4 minute following the acid treatment and the shed matriptase accumulated rapidly (Fig 4B, lower panel). These kinetics indicates that matriptase zymogen activation is followed very closely by matriptase shedding and the two regulatory processes appear to occur almost simultaneously.

**Fig 5:**
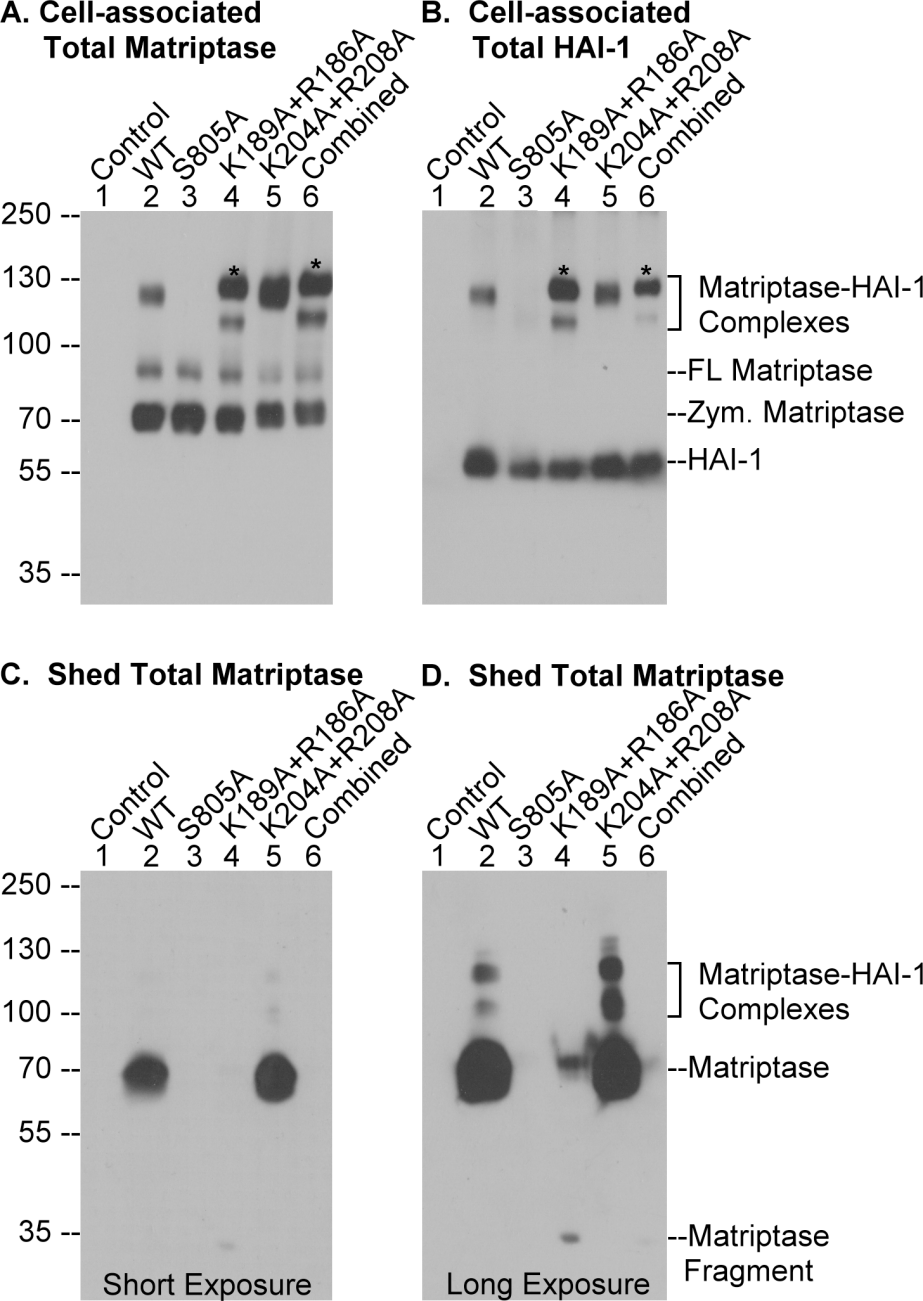
Matriptase shedding depends on matriptase zymogen activation and cleavage at Arg or Lys residues in the link region between the SEA and the CUB domain. Wild-type (lanes 2, WT) or matriptase mutants with point mutation at Ser-805 (lanes 3, S805A), mutations at Lys-189 and Arg-186 together (lanes 4, K189A+R186A), mutations at Lys-204 and Arg-208 together (lanes 5, K204A+R208A), or mutations at all four positively charged amino acid residues together (lanes 6, combined) were expressed in 293T HEK cells. The parental cells (lanes 1, Control) and the transfected cells were exposed to a pH 6.0 buffer to induce matriptase zymogen activation. The cell lysates (*A*. and *B*. Cell-associated) and the conditioned buffers (*C*. and *D*. Shed) were analyzed by immunoblot under non-reducing and non-boiled conditions for matriptase species (*A*. *C*. and *D*., Total Matriptase) and HAI-1 species (*B*. Total HAI-1). In order to detect the trace matriptase-HAI-1 complexes and other matriptase species shed, the immunoblots were analyzed with both shorter exposure (*C*.) and longer exposure (*D*.). The shed fractions were concentrated to the same volume as that of the cell lysate. Equal volume of the cell lysate and the shed fraction were subjected to SDS-PAGE. Matriptase and HAI-1 species are indicated. FL stands for full-length and Zym for zymogen. The matriptase-HAI-1 complexes with slightly greater mass are indicated with *. The data are representative of more than 3 independent experiments.

Given the involvement of de novo proteolytic cleavage in matriptase shedding, the close temporal relationship suggests that matriptase shedding might depend on matriptase zymogen activation and involve matriptase proteolytic activity directly or indirectly through the activation of downstream substrate proteases. To test the role of matriptase zymogen activation in matriptase shedding, we compared the shedding of wild-type matriptase to that of a variant with a point mutation at Ser-805 in the active site triad. As described above and previously [10], matriptase undergoes autoactivation to acquire its enzymatic activity using its intrinsic zymogen activity and so mutation at the active site triad Ser-805 will prevent matriptase autoactivation. The cognate matriptase inhibitor, HAI-1, was co-expressed with these matriptase species due to the essential role HAI-1 plays in normal matriptase expression, intracellular trafficking, and zymogen activation [1,40]. When co-expressed with HAI-1, wild-type matriptase underwent zymogen activation, resulting in the formation of the 120-kDa matriptase-HAI-1 complex in response to transient exposure to a pH 6.0 buffer (Fig 5A and 5B, lanes 2). Consistent with natural endogenous matriptase and HAI-1, matriptase shedding also occurred (Fig 5C, lane 2). Very low levels of matriptase-HAI-1 complexes of 110- and 95-kDa were also shed and were detected only after longer exposure in immunoblot analysis (Fig 5D, lane 2). In contrast to the wild-type matriptase, the Ser-805 to Ala mutant matriptase not only lost the ability to undergo autoactivation in response to transient acidification (Fig 5A and 5B, lanes 3) but was also not shed from the cells (Fig 5C and 5D, lanes 3). Collectively, these data suggest that matriptase shedding is dependent on and tightly coupled with matriptase zymogen activation.

### Matriptase shedding requires proteolytic cleavage at positively charged amino acid residues, primarily Arg-186, between the SEA and the first CUB domain

Since we suspected that matriptase might directly mediate the proteolytic cleavage required for its own shedding, we next examined if the cleavage occurred at positively charged amino acid residues involving a tryptic activity. There are two lysine residues, Lys-189 and Lys-204, and two arginine residues, Arg-186 and Arg-208, present in the junction of the SEA domain and the first CUB domain in matriptase. We first used site-directed mutagenesis to generate expression constructs in which these four amino acid residues were mutated in three groups: 1) Arg-186→Ala plus Lys-189→Ala; 2) Lys-204→Ala plus Arg-208→Ala; and 3) the combination of all four residues mutated to Ala. When these matriptase variants were co-expressed with HAI-1 in HEK-293T cells we found that like wild-type matriptase they underwent zymogen activation followed by HAI-1 inhibition of the newly generated active matriptase when the cells were transiently exposed to a pH 6.0 buffer (Fig 5A and 5B, lanes 4–6). While mutations of the four amino acids had no impact on matriptase autoactivation, matriptase shedding was, however, almost completely suppressed when Arg-186 and Lys-189 were mutated to Ala (Fig 5C, lane 4). A very low level of matriptase shedding was detectable but only after very long exposure of the immunoblots (Fig 5D, lane 4). Mutation of Lys-204 and Arg-208 did not affect matriptase shedding (Fig 5C and 5D, lanes 5). Not surprisingly, matriptase shedding was also blocked when all four positively charged amino acids were mutated (Fig 5C and 5D, lanes 6). This analysis implicates Arg-186 and/or Lys-189 as likely the candidate residues at which the cleavage responsible for matriptase shedding occurs.

**Fig 6:**
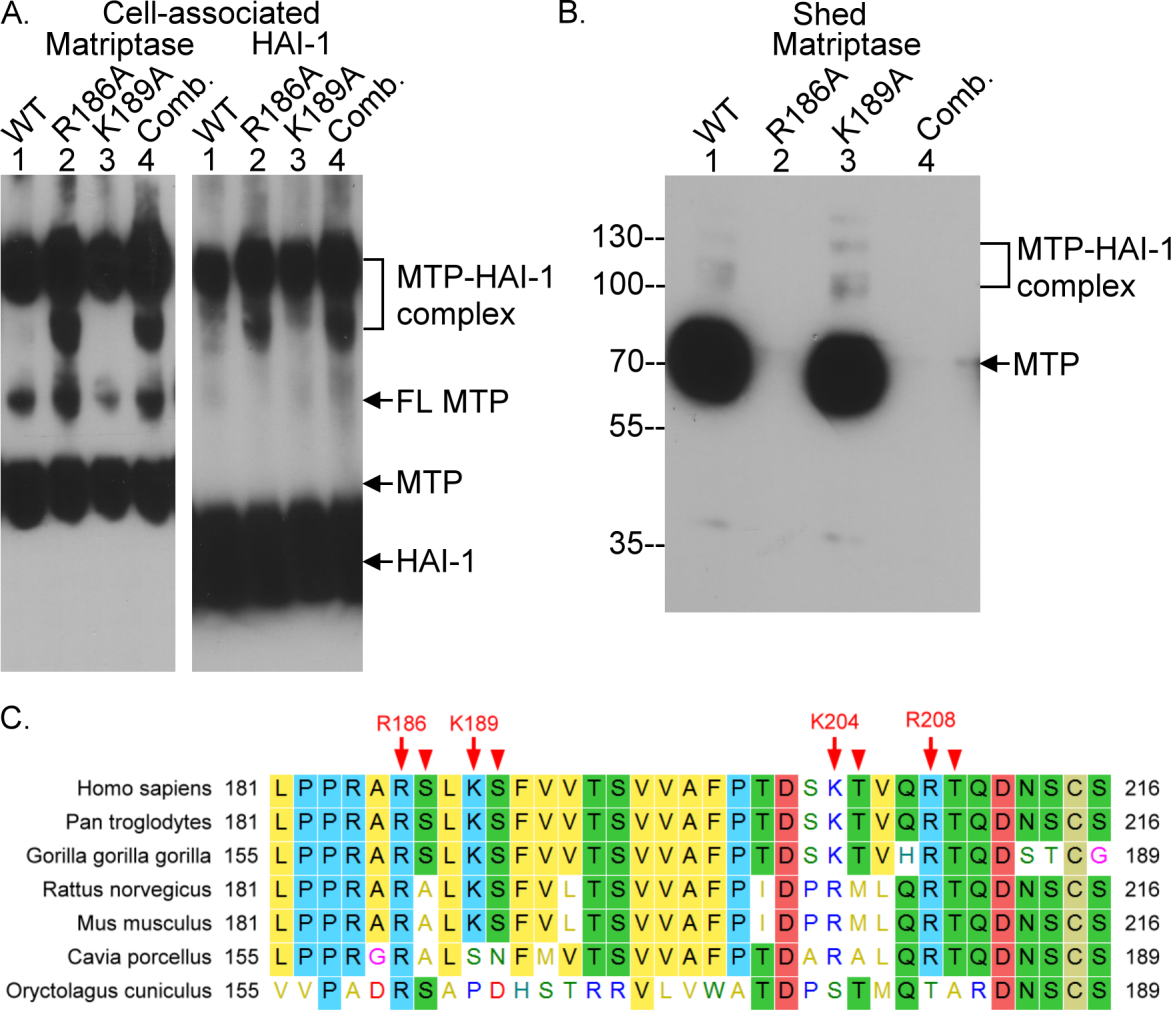
Arg-186 is the cleavage site responsible for matriptase shedding following matriptase zymogen activation. The wild-type (lanes 1, WT) and matriptase mutants, including point mutation at Arg-186 (lanes 2, R186A), point mutation at Lys-189 (lanes 3, K189A), or mutations at Arg-186 and Lys-189 together (lanes 4, comb.) were expressed in 293T HEK cells. The cells were exposed to a pH 6.0 buffer to induce matriptase zymogen activation. The cell lysates (*A*. Cell-associated) and the conditioned buffers (*B*. Shed) were analyzed by immunoblot under non-reducing and non-boiled conditions for matriptase species (*A*. and *B*, Matriptase) and HAI-1 species (*A*. HAI-1). Matriptase and HAI-1 species are indicated. MTP stands for matriptase and FL for full-length. The shed fractions were concentrated to the same volume as that of cell lysate. Equal volume of the cell lysate and the concentrated were subjected to SDS-PAGE. *C*. The amino acid sequences of the linker region between the SEA domain and the CUB domain of matriptase are compared among various species, including Homo sapiens (human, Swiss-Prot accession no. Q9Y5Y6), Pan troglodytes (chimpanzee, Swiss-Prot accession no. H2Q547), Gorilla gorilla gorilla (gorilla, Swiss-Prot accession no. G3S550), Rattus norvegicus (rat, Swiss-Prot accession no. Q9JJI7), Mus musculus (mouse, Swiss-Prot accession no. P56677), Cavia porcellus (Guinea pig, Swiss-Prot accession no. H0VJD4), and Oryctolagus cuniculus (rabbit, Swiss-Prot accession no. G1SX25). The full protein sequences were obtained from Uniprot. Sequences were aligned by Molecular Evolutionary Genetics Analysis version 7.0 for bigger datasets using the MUSCLE method [41]. The data are representative of at least 3 independent experiments.

Using the same approach, constructs with a single mutation at Arg-186 or Lys-189 were generated and analyzed, allowing us to demonstrate that Arg-186 is the cleavage site responsible for matriptase shedding. Matriptase variants with a point mutation at Arg-186 and/or Lys-189 can undergo acid-induced autoactivation, followed by rapid HAI-1 inhibition similar to wild-type matriptase (Fig 6A). Matriptase shedding, however was completely blocked by point mutation at Arg-186 but not Lys-189 (Fig 6B, lanes 2 and 3). Matriptase shedding was also suppressed in the construct with the combined mutation at Arg-186 with Lys-189 (Fig 6B, lane 4). When the matriptase amino acid sequence flanking Arg-186 was aligned with the analogous sequences from the matriptase orthologs from a variety of different species (Fig 6C), the shedding motif with the amino acid sequence PRAR was found to be highly conserved among primates, rodents, and the guinea pig, but not the rabbit. The PRAR shedding motif perfectly matches the matriptase cleavage preference, which includes positively charge amino acid residues as the P1 site, amino acid with non-polar small side chain as the P2 site, and positively charge amino acids at the P3/P4 site [14]. The match between the shedding motif and the matriptase cleavage preference, the requirement of matriptase zymogen activation for matriptase shedding, and the tightly coupled kinetics of zymogen activation and shedding strongly support the role of matriptase proteolytic activity in matriptase shedding.

### The activated matriptase present in cell-associated matriptase-HAI-1 complexes has been cleaved at Arg-186

**Fig 7:**
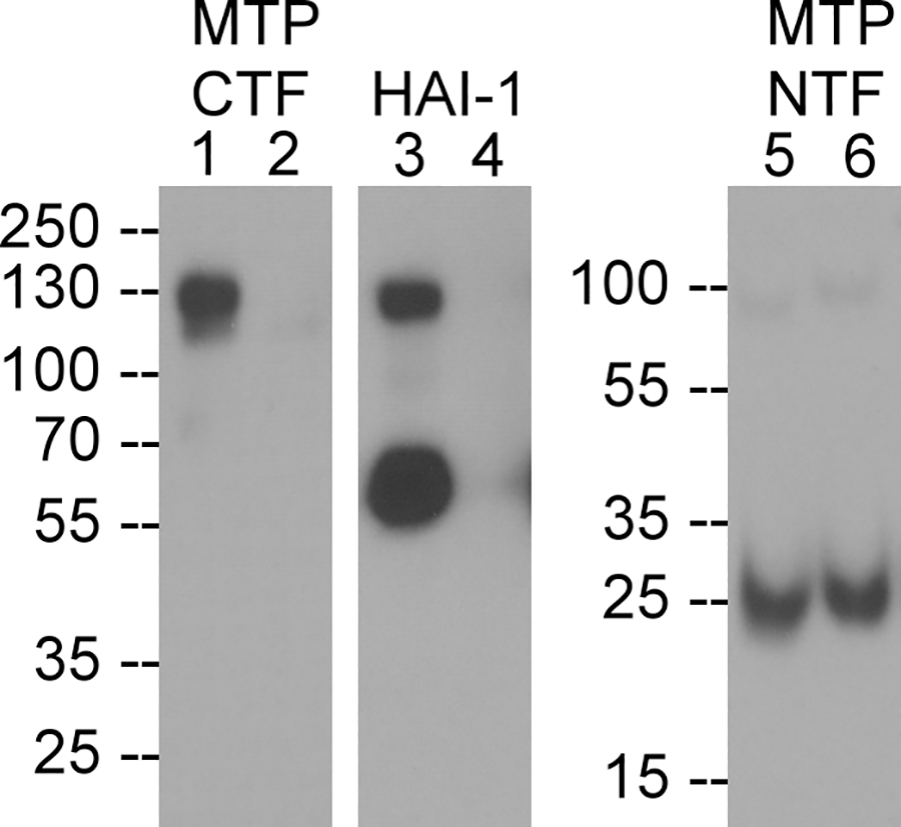
Matriptase in complex HAI-1 is a shed species. HaCaT human keratinocyte cells were induced to activate matriptase by pH 6.0 buffer treatment. Matriptase-HAI-1 complex in the lysate was immunodepleted with HAI-1 mAb-Sepharose. The cells lysate (lanes 1, 3, and 5) and the immunodepleted fraction (lanes 2, 4, and 6) were analyzed by western blot under non-reducing and non-boiled conditions for matriptase CTF species (lanes 1 and 2), HAI-1 species (lanes 3 and 4), and matriptase NTF (lanes 5 and 6). MTP stands for matriptase, CTF for C terminal fragment, and NTF for N terminal fragment. The data presented are representative of at least 2 independent experiments.

We noted that the cell-associated HAI-1 complexes formed with matriptase variants in which mutation at Arg-186 prevented matriptase shedding (indicated by * in Fig 5), appeared to be slightly larger than the HAI-1 complexes with wild-type matriptase or with matriptase variants that did not block matriptase shedding (Fig 5). We reasoned that this size difference was the result of the lack of cleavage at Arg-186 in those mutants in which shedding was blocked, resulting in a larger matriptase-HAI-1 complex. If true, this would imply that the activated, cell-associated wild-type matriptase, had undergone cleavage at Arg-186 and so its association with the lipid bilayer was dependent on its interaction with the membrane-bound HAI-1. To test this hypothesis, the cell-association status of the NTF and CTF of the activated (wild-type) matriptase complexed with HAI-1 was determined by a combination of immunodepletion of the 120-kDa (activated) matriptase-HAI-1 complex using the HAI-1 mAb M19 followed by immunoblot analysis for the matriptase NTF using the mAb PS6. As shown in Fig 7, the 120-kDa matriptase-HAI-1 complex was detected by the matriptase CTF mAb (Fig 7, lane 1) and the HAI-1 mAb (Fig 7, lane 3) but not the matriptase NTF mAb (Fig 7, lane 5). Incubation of the cell lysate with HAI-1 mAb M19-Sepharose depleted the 120-kDa matriptase-HAI-1 complex (Fig 7, lane 2) as well as the uncomplexed HAI-1 (Fig 7, lane 4). The 25-kDa matriptase NTF, however, was not depleted along with the 120-kDa matriptase-HAI-1 complex and remained in the cell lysate (Fig 7, comparing lane 6 with lane 5), indicating that the CTF of the cell-associated activated matriptase has been cleaved from the matriptase NTF. The presence of the 25-kDa matriptase NTF in the unbound fraction addresses the specificity of immunodepletion. These data confirmed that the matriptase in the cell-associated 120-kDa matriptase-HAI-1 complex has been cleaved at Arg-186.

## Discussion

Many lines of evidence provided in current study support the idea that matriptase shedding is an autonomous process which involves de novo proteolytic cleavage, and which depends on and is closely temporally coupled with matriptase zymogen activation. This autonomous mechanism has the potential to prolong the active half-life of enzymatically active matriptase in the face of the abundant and potent endogenous inhibitor HAI-1 present at similar sub-cellular loci. The shed active matriptase could thereby gain more access to substrates in the extracellular milieu. The involvement of de novo proteolytic cleavage is supported by the smaller size of the shed matriptase species compared to the cell-associated counterparts and the identification of Arg-186 as the cleavage site; the mutation of which abolishes matriptase shedding. The lack of shedding of the matriptase variant that cannot undergo zymogen autoactivation provides evidence for the dependence of shedding on matriptase zymogen activation, which indicates that the two events are tightly coupled. The perfect match of the sequences flanking Arg-186 to the matriptase cleavage preference, in conjunction with the dependence of shedding on zymogen activation, which generates enzymatically active matriptase, supports the role of matriptase proteolytic activity in matriptase shedding. While the possibility remains that the cleavage responsible for matriptase shedding is mediated by a downstream protease activated by matriptase, the direct role of matriptase activity in matriptase shedding appears to be the most likely scenario, particularly in the light of the rapid kinetics that match the kinetics of matriptase zymogen activation and inhibition of the nascent active matriptase by HAI-1.

The rapid proteolytic cleavage at Arg-186 following matriptase zymogen activation also occurs to a proportion of the activated matriptase which remains cell-associated through the formation of a complex with membrane-associated HAI-1 (Fig 7). It remains unclear whether the formation of this complex occurs before or after the cleavage at Arg-186. The membrane-associated activated matriptase is, nevertheless, tethered on the cell membrane via the transmembrane domain of its complex partner HAI-1, which results in inactivation of the nascent active matriptase. As a result, while activated matriptase can remain cell-associated, its enzymatic activity is inhibited by HAI-1 with the result that cell-associated free, active matriptase is a very short-lived species. Thus, enzymatically active matriptase can have a longer life only by shedding into the extracellular milieu, consistent with our previous study in which moderate levels of matriptase activity can be detected in the conditioned media of cells but not in cell lysates [21]. The presence of enzymatically active matriptase in the extracellular milieu but not on the plasma membrane shifts matriptase function to that of a pericellular protease rather than a membrane-associated one, even though matriptase is synthesized as a transmembrane protein. As a pericellular protease, matriptase could gain access to more substrates than as a membrane-associated protease whereas binding to the plasma membrane for some substrates, such as uPA, could significantly enhances the matriptase-mediated activation [22]. The lack of enzymatically active matriptase on the cell surface is at odds with several previous studies in which enzymatically active matriptase has been reported to be cell-associated [6,42–47]. Given that the cleavage at Arg-186 deprives active matriptase of its membrane anchorage, it is unclear what could be the mechanism by which active matriptase could be associated with the cell membrane and remain enzymatically active as reported in these studies.

Small amounts of activated matriptase-HAI-1 complex were shed along with induction of matriptase zymogen activation (Figs 5 and 6). In our previous study, the majority of the cell-associated activated matriptase-HAI-1 complex was shed 1–6 hours after the cells were return to a physiological pH [19]. Matriptase is expressed and targeted to the basolateral plasma membrane of polarized epithelial cells both *in vitro* in cultured cells and *in vivo* in epithelial tissues [48,49]. Matriptase-HAI-1 complex was, however, shed from the apical plasma membrane as demonstrated in differentiated Caco-2 cells [48] which is consistent with the fact that matriptase-HAI-1 complexes are secreted in human body fluids, including milk, semen and urine [16,48]. In contrast to activated matriptase-HAI-1 complex, matriptase zymogen is shed only from the basolateral plasma membrane. For the secretion of matriptase-HAI-1 complex, the polarized cells must internalize the matriptase-HAI-1 complex and translocate the complex to the apical plasma membrane for secretion, a process known as transcytosis. The differential shedding of matriptase zymogen versus the activated matriptase-HAI-1 complex in polarized epithelial cells could result from the fact that HAI-1 but not matriptase can undergo transcytosis in polarized Caco-2 cells [49]. It is worth noting that the N-termini identified for the shed matriptase purified from human milk are Ser-190 or Thr-205, rather than Ser-187 [31]. The difference in the cleavage sites, Arg-186 versus Lys-189 and Lys-204, suggests that further cleavage of activated matriptase-HAI-1 complex might occur in the subsequent processes, including internalization, transcytosis, and secretion. Alternatively, the discrepancy could be the result of differences in the mechanisms involved in the shedding of activated matriptase in the more physiologically relevant *in vivo* context versus the shedding of matriptase zymogen *in vitro* in cultured cells. The matriptase species isolated from human milk is activated matriptase in complexes with HAI-1, whereas the species detected in the conditioned medium or buffer is primarily matriptase zymogen. The zymogen activation and/or the complex formation with HAI-1 might affect the exposure of the potential cleavage sites and subsequent selection of shedding cleavage sites. Nevertheless, the link region between the SEA domain and the CUB domain contains four tryptic cleavage sites Arg-186, Lys-189, Lys-204 and Arg-208, all of which are followed by residues with hydroxyl group in their side chains, such as serine and threonine (Fig 6C). Furthermore, Arg-186 is preceded by Ala-185 and Arg-184, and Lys-189 is by Leu-188 and Arg-186. The sequences prior to Arg-186 and Lys-189 place both residues in the context of perfect shedding cleavage sites matched to the matriptase cleavage preference. It is also worth noting that the entire link region, including the four putative cleavage sites, is almost identical among human, chimpanzee, and gorilla, and is highly conserved between primates and rodents with the prominent variation in the amino acid residues following the putative cleavage sites, such as the substitution of the Ser-187and Thr-205 in human with Ala and Met in rodent (Fig 6C).

Although the calculated mass of the matriptase NTF is 16-kDa, the apparent size on SDS-PAGE is roughly 25-kDa. This size difference could be attributed to the formation of anomalous detergent-protein complexes. It has been demonstrated that SDS loading and protein helicity affect SDS-PAGE migration of an artificial helix-loop-helix (hairpin) structure derived from human cystic fibrosis transmembrane conductance regulator [50]. SDS is able to aggregate around and encase alpha-helices in SDS-micelles to form atypical detergent-protein complexes that results in a gel shift. The structure of matriptase NTF is predicted by Iterative Threading Assembly Refinement (I-TASSER) [51] to resemble a helix-coil-helix structure, which includes the two alpha-helices of the transmembrane domain and the N-terminal half of the SEA domain at both ends and a random coil in the between. Furthermore, the apparent size of matriptase NTF remains at 25-kDa under boiled and reducing conditions, excluding the possibility that the unexpected size is caused by the interaction and complex formation of matriptase NTF with itself or another protein. The fact that a smaller minor band with predicted size around 15-kDa coexists with the 25-kDa major species is consistent with the hypothesis that the matriptase NTF has a propensity to form atypical detergent-protein complexes.

Matriptase zymogen, like many other serine protease zymogens, possesses weak intrinsic enzymatic activity. Such intrinsic activity is typically several orders of magnitudes less than that of the proteolytic activated enzyme. The intrinsic activity of serine protease zymogen was initially detected by the formation of stable covalent adducts of protease zymogens with active-directed inhibitors, such as diisopropyl fluorophosphate (DFP)[52]. Reaction rates for protease zymogens are 10^4^–10^5^ times slower than those of their active counterparts. Quantification of the exact magnitude of matriptase zymogen intrinsic activity and comparison of the rate with that of cleaved, active matriptase presents a real challenge due to the fact that matriptase zymogen undergoes autoactivation to become an active enzyme. Substrate selection would be the first problem in establishing such an assay. For example, if an optimal matriptase fluorogenic peptide substrate were selected to monitor matriptase zymogen intrinsic activity, any nascent active matriptase, even if present at extremely low levels, would cleave the substrate at a much higher rate than the matriptase zymogen. There is no obvious way to distinguish zymogen-mediated from active matriptase-mediated cleavage of an optimal active matriptase substrate. Furthermore, serine protease zymogens contain distorted substrate binding pocket [53] and typically have very different substrate selectivity and exhibit different cleavage preferences compared to their active protease counterparts. For example, the best substrate for chymotrypsinogen was shown to be Boc-Ala-nitroaniline and Z-Gly-Hyp-Gly-nitroaniline for trypsinogen [54]. The P1 sites of these zymogen substrates are very different from that of the selective substrates for chymotrypsin and trypsin. This important concern and difficulty with the monitoring matriptase zymogen intrinsic activity does not appear to have been fully addressed in the study of Inouye et al [55], in which Ac-KTKQLR-7-amino-4-methylcoumarin (AMC) was used as the substrate to monitor both matriptase zymogen and active matriptase activity, based on its sequence similarity to that flaking the activation motif of matriptase. The 48 hour incubation time used for monitoring substrate cleavage by matriptase zymogen strongly suggest that the substrate selection and/or the experimental design in the Inouye et al., study were likely suboptimal. A similar concern is the 8 hour incubation time employed to determine the kinetic parameters of active matriptase. More concerning is the fact that the data presented show almost no difference in the k_cat_, K_m_, and k_cat_ / K_m_ values for active matriptase between pH 7.5 and pH 6.0 which is not consistent with the pH-dependence of the catalytic reactions of serine protease at alkaline pH and the bell-shaped pH-rate (k_cat_/K_m_) profile [56]. Ionization of the imidazole group of the His residue in the catalytic triad and the α-NH_2_ group of the non-polar small amino acid residue at the NH_2_-terminus of the heavy chain of serine protease is important in the catalytic reaction of serine proteases. For serine protease zymogen activity, the ionization of the α-NH_2_ group of the non-polar small amino acid residue at the NH_2_-terminus of the heavy chain should not occur, because it is participating in a peptide linkage. Thus, zymogen intrinsic activity is a completely different concept that should not be compared or confused with that of active serine protease.

Our conclusions that matriptase enzymatic activity is involved in its own shedding, that cleavage occurs at Arg-186, and that shedding is temporally closely coupled with zymogen activation are at considerable odds with the studies published by Drs. Fushiki and Inouye of Kyoto University [57–59]. Indeed, the data presented herein have strengthened our long-held concern that they may in fact be studying a different protease. Several reasons have led to this concern. Firstly, the molecule they refer to as matriptase is not reliably detected in the skin [57], whereas the rest of the field consistently report expression in the skin which indeed is the tissue most impacted in matriptase knockout mice [60] and in patients with matriptase mutations [7,8]. While in one of their papers matriptase was predominantly detected on the basolateral plasma membrane [57], which is similar to the observations of ourselves and other investigators, in their other paper, no matriptase was detected on either the basolateral or apical surface of the cells [59]. Interestingly, matriptase was detected in the conditioned medium from these cells. The subcellular distribution reported in this latter study would suggest that the molecule they describe as matriptase is a secreted rather than a membrane-bound protease. In the same study, when Gly-149 was mutated, which generates full-length matriptase, the protease suddenly becomes a membrane-bound protein that is primarily targeted to the basolateral membrane with no shed matriptase being detected. This should have led to the conclusion that the Gly-149 mutation results in the association of the protease with the cell membrane. When the matriptase serine protease domain was deleted, the non-catalytic domains also become membrane-associated, mainly targeted to the basolateral plasma membrane, but were also, surprisingly secreted from the apical surface. In another paper from this group [58], mutation of Ser-805 in the active site triad, the mutant became much less membrane-associated than wild-type matriptase. This should have led them to conclude that the matriptase active site triad is important for membrane-association, however, they just simply ignore the change in the subcellular distribution profile associated with the mutation at the active site triad. The internally inconsistent nature of the data presented in the publications from this group, and the conclusions drawn that do not appear consistent with the data they present, leads us to conclude that the work is potentially problematic and so, for the sake of clarity, we have chosen not to address the apparent discrepancy between the work presented here and the studies mentioned.

**Fig 8:**
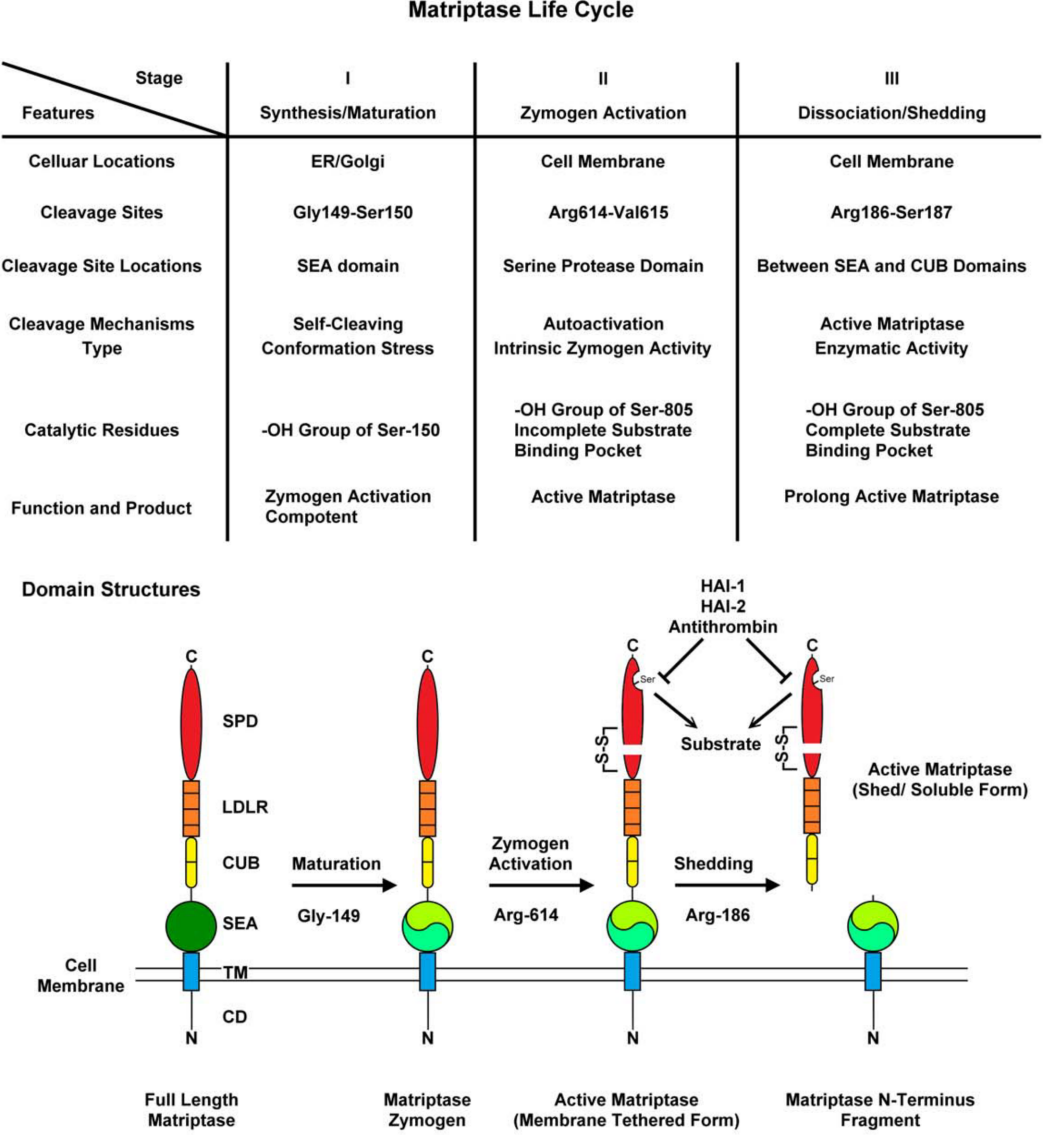
The summary and schematic illustration of matriptase life cycle. The matriptase life cycle has three distinct stages which are summarized by schematic illustrations of the changes in the protein domain structures. A description of matriptase life cycle can be found in the Discussion. SPD stands for the serine protease domain; LDLR for the LDL receptor class A domain; CUB for the CUB (complement C1r/C1s, Uegf, Bmp1) domain; SEA for the SEA (sea urchin sperm protein, enterokinase and agrin) domain; TM for the transmembrane domain; CD for the cytoplasmic domain; N for the N-terminus; and C for the C-terminus.

In summary, the discovery of the role of matriptase proteolytic activity in its own shedding adds important information about a part of the matriptase life cycle, which features three distinct peptide bond cleavages mediated via different cleavage mechanisms at different regulatory stages. Matriptase is synthesized and undergoes maturation in the endoplasmic reticulum and Golgi apparatus. The first peptide bond cleavage at Gly149-Ser150 in the middle of the SEA domain occurs spontaneously during synthesis and maturation. Conformation stress and the conserved hydroxyl group of Ser-150 are responsible for the self-cleaving mechanism, resulting in a cleaved SEA domain, the fragments of which remains tightly associated via a thermodynamically stable non-covalent interaction. The mature matriptase zymogen is, therefore, composed of two chains and is tethered on the cell membrane via the tightly associated SEA domain and the transmembrane domain. The mature matriptase zymogen with a cleaved SEA domain is competent and ready to respond to the stimuli that trigger matriptase autoactivation, which requires a second peptide bond cleavage. The zymogen activation cleavage occurs between Arg-614 and Val-615 at the junction between the non-catalytic domains and the catalytic domain, which remain held together by a disulfide linkage after autoactivation. Intrinsic matriptase zymogen activity involving the conserved hydroxyl group of Ser-805 is responsible for the zymogen activation cleavage although the substrate binding pocket is not yet well-formed. Immediately following zymogen activation, three different events can occur rapidly and almost simultaneously to the newly generated active matriptase: acting on its substrates, inhibition by HAI-1 binding, and/or cleavage and release of matriptase from the cell surface. The last cleavage in the matriptase life cycle primarily between Arg-186 and Ser187 is likely catalyzed by the conserved hydroxyl group of Ser-805 of active matriptase in which the mature substrate binding pocket has been formed by the process of zymogen activation. The peptide bonds cleaved are at the junction linking the SEA domain and the first CUB domain, which results in the shedding of the bulk of matriptase extracellular domains, including the catalytic domain. The matriptase N-terminal fragment, comprised of the intracellular domain, transmembrane domain, and the entire SEA domain, remains on the cell surface and its fate has yet to be elucidated. The matriptase life cycle is summarized in Fig 8.
